# On the design of linked datasets mapping networks of collaboration in the genomic sequencing of *Saccharomyces cerevisiae*, *Homo sapiens*, and *Sus scrofa*

**DOI:** 10.12688/f1000research.18656.2

**Published:** 2020-10-23

**Authors:** Mark Wong, Rhodri Leng

**Affiliations:** 1 Urban Studies, School of Social and Political Sciences, University of Glasgow, Glasgow, G12 8QQ, UK; 2 Science, Technology and Innovation Studies, University of Edinburgh, Edinburgh, EH1 1LZ, UK

**Keywords:** Bibliometrics, Bibliographic Database, network analysis, genomics, S. cerevisiae, Homo sapiens, Sus scrofa, history of science

## Abstract

This paper describes a unique two-step methodology used to construct six linked bibliometric datasets covering the sequencing of *Saccharomyces cerevisiae*, *Homo sapiens*, and S *us scrofa *genomes. First, we retrieved all sequence submission data from the European Nucleotide Archive (ENA), including accession numbers associated with each species. Second, we used these accession numbers to construct queries to retrieve peer-reviewed scientific publications that first linked to these sequence lengths in the scientific literature. For each species, this resulted in two associated datasets: 1) A .csv file documenting the PMID of each article describing new sequences, all paper authors, all institutional affiliations of each author, countries of institution, year of first submission to the ENA, and the year of article publication, and 2) A .csv file documenting all institutions submitting to the ENA, number of nucleotides sequenced, number of submissions per institution in a given year, and years of submission to the database. In several upcoming publications, we utilise these datasets to understand how institutional collaboration shaped sequencing efforts, and to systematically identify important institutions and changes in network structures over time. This paper, therefore, should aid researchers who would like to use these data for future analyses by making the methodology that underpins it transparent. Further, by detailing our methodology, researchers may be able to utilise our approach to construct similar datasets in the future.

## Introduction

This paper describes the methodology used to construct six novel datasets for the European Research Council funded project, *Medical Translation in the History of Modern Genomics*; a project exploring the history of scientific collaboration around DNA sequencing. The datasets contain information specific to the genomic sequencing of *Saccharomyces cerevisiae* (baker’s yeast), *Homo sapiens* (human), and *Sus scrofa* (domestic pig), and consist of data related to sequence submissions to public databases and co-authorship relations underpinning the description of those sequences in the scientific literature. As part of this project, we have stored all relevant datasets in the data repository at the University of Edinburgh (see *Data availability*; Wong *et al*., 2019).

In what follows, we first describe a unique two-step methodology that involved:

1. Extracting data on sequence submissions to the European Nucleotide Archive (ENA a public, open access database) via automated routines and Application Programme Interfaces (APIs).
2. Linking particular sequence submissions to peer-reviewed publications that first described these in the literature via API queries, which utilised sequence accession numbers to mine Europe PubMed Central and SCOPUS.

We then discuss our approach to re-structuring and cleaning these data and offer a description of the content of each dataset. Finally, we reflect on the strengths and weaknesses of these datasets and methods.

## Materials and methods

### Data collection

This project entailed a large and unique data collection exercise of over 13 million records, which were retrieved via 30 million API queries to three different databases. This involved a two-step process. First, we retrieved all sequence submission data from the ENA, including accession numbers associated with particular sequence lengths. Second, we used these accession numbers to construct API queries to retrieve peer-reviewed scientific publications that first discussed and linked to these sequence lengths in the scientific literature. 

### Extracting ENA submission data

We retrieved sequence submission data from the ENA for each of the three species over defined periods – *S. cerevisiae* (1980–2000), *H. sapiens* (1985–2005), and *S. scrofa* (1990–2015). The date ranges for each species were selected based on the history of science objectives underlying our project. The purpose was to capture submissions before, during, and after the completion of concerted efforts to systematically sequence the genome of each of the organisms. The search was conducted by making a series of calls to ENA’s API for each species and each year the study investigated. The query was constructed by specifying the taxon’s number (tax_eq) in the ENA index (i.e. 9606 for *H. sapiens*, 4932 for *S. cerevisiae* and 9823 for *S. scrofa*) and the sequence release date (first_public) to filter records that were released within a certain year. The search parameter of first_public was specified as “greater than or equal to” 1 ^st^ January and “less than or equal to” 31 ^st^ December of the year. Additional parameters were used to specify search for sequence release records ( result=sequence_release) and download the data in .XML format ( display=xml). In cases where records per year exceeded the ENA’s limit of 100,000 records per API call, the pagination function ( offset) was deployed.

This procedure allowed us to mine the ENA database based on the species and years relevant to our study and extracted data on: 1) the number of nucleotides submitted for each of these species; 2) all accession numbers associated with these sequence lengths; 3) the date of submission; 4) the name of the submitting individual and their institutional affiliation (if available); and 5) papers in the scientific literature associated with each accession number (if specified by the submitter) ( Li *et al*., 2015). Further details about the API queries are contained in the R scripts made available together with the datasets (see *Software availability*; UofGMarkWong, 2019). As the ENA is part of The International Nucleotide Sequence Database Collaboration (INSDC), which facilitates the sharing of information of three main sequence databases, including the European Nucleotide Archive, based in the European Bioinformatics Institute, GenBank, provided by the US National Centre for Biotechnology Information (NCBI), and DNA Data Bank of Japan (DDBJ), we were able to retrieve all sequence submissions from institutions participating in these databases. Once collected, we utilised the R statistical environment ( R Development Core Team, 2016) to structure these data for further cleaning and analysis. In Table 1, we report the total records retrieved via this process.

**Table 1.** Total of ENA accession numbers and Europe PMC publication records retrieved for *S. cerevisiae*, *Homo sapiens*, and *Sus scrofa*.

*Species*	Total ENA submissions/ accession numbers (nucleotides)	Accession numbers that contain submitter records (nucleotides)	Accession numbers that contain publication records (nucleotides)	Accession numbers in which submitter or publication records were not found (nucleotides)	PMIDs retrieved from Europe PMC
*S. cerevisiae* *(1980 – 2000)*	18,521 (32,726,254)	5,421 (22,967,465)	3,343 (11,517,249)	10,875 (5,898,748)	3,158
*Sus scrofa* *(1990 – 2015)*	3,322,337 (18,890,916,045)	1,676,935 (10,275,568,002)	1,435,419 (2,969,582,95)	338,890 (8,174,825,230)	2,464
*Homo sapiens* *(1985–2005)*	10,091,109 (21,034,707,659)	2,619,237 (16,942,665,389)	2,582,496 (10,466,788,214)	5,055,436 (3,996,992,385)	33,910

### Extracting Europe PMC and SCOPUS publication data

However, as the availability of submitter information was found to be sparse and the ENA often lists one submitting institution only (see Table 1), we used publication data as a proxy to identify collaboration between institutions. We generated queries to the Europe PubMed Central’s (Europe PMC) API by using the ENA accession number as a parameter to search for associated publications ( EMBL_PUBS). This linkage allowed us to identify a list of PubMed IDs (PMIDs) of the publications linked to these accession numbers ( Lopez *et al*., 2014). In addition, other parameters were used including result_type=core to return full metadata available, format to download as .JSON files, cursorMark as a pagination option, and the default result limit of each API call ( pageSize) at 1000 publication records. We deployed a routine to automate the search for each accession number in our dataset. The routine’s procedure to compose and make an API call to Europe PMC using a list of accession numbers (pre-extracted using the ENA API call detailed) have been made available in an online repository (see *Software availability*; UofGMarkWong, 2019).

We then extracted fuller data on all authors, their institutional affiliations, the city and country of institution and the date of publication in SCOPUS using the PMIDs as a search parameter ( PMID) and utilising other default parameters such as apikey, apart from view=complete to specify returning of full meta-data. The routines and R scripts used are also available (see *Software availability*; UofGMarkWong, 2019). The use of two bibliometric databases was considered necessary, as while EuropePMC allowed searches for publications linked to and specifically describing an accession number, it only holds institutional information of the corresponding author for all publications published before 2014 ( Europe PMC Consortium, 2015). SCOPUS holds fuller bibliometric records of all authors and their institutions, particularly for biomedical and natural science literature ( Rotolo & Leydesdorff, 2015). This was crucial for mapping institutional collaboration. However, this database only allows searches based on its text-mining functions and returns publications that mention an accession number anywhere in the text – thus the necessity of inputting the PMIDs retrieved via EuropePMC.

We selected only the first articles to be published associated with an accession number because first publications are more likely to be written by the groups responsible for the submission of the original version of the sequence (either to describe their contribution or to use it in agricultural or biomedical research). Although this correspondence between submission and first publication is not universal, our search strategy excluded papers that utilised particular sequence lengths that had already been described in the literature and consequently refined our corpus of PMIDs (see differences between Table 1 and Table 2).

**Table 2.** Total number of PMIDs, institutions, and countries extracted from SCOPUS for *S. cerevisiae* (1980–2000), *Homo sapiens* (1985–2005) and *Sus scrofa* (1990–2015).

Species	PMIDs	Institutions	Countries
*S. cerevisiae* (1980–2000)	2,887	685	42
*Sus scrofa* (1990–2015)	1,947	1,272	63
*Homo sapiens* (1985–2005)	24,726	6,014	102

### Data cleaning and description

Once collected, researchers in our team cleaned these datasets via VantagePoint (2017) v.10 by using a combination of fuzzy logic algorithms available in the software (i.e. “Fuzzy word matching” to make fuzzy word comparisons at 95% or lower) and manual cleaning to standardise institution, author and country names according to a pre-specified protocol. The protocol specified and ensured consistency in name conventions, fully spelling out acronyms and abbreviations, removing articles and legal entities, using proper case conventions, removal of white spaces and ineligible characters, removal of duplicates, and keeping school and department data if it appears more than 50 times in the dataset. Missing data, particularly regarding institutional affiliation, was filled manually by scrutinising the record on SCOPUS’ web front-end. To replicate this cleaning process, other open source software, such as OpenRefine, may also be used as an alternative.

In total, each species has two associated datasets: 1) A .csv file documenting the PMID of each article describing new sequences, all paper authors, all institutional affiliations of each author, countries of institution, year of first submission to the ENA, and the year of article publication, and 2) A .csv file documenting all institutions submitting to the ENA, number of nucleotides sequenced, number of submissions per institution in a given year, and years of submission to the database.

The ENA dataset documents the volume of DNA sequencing per year and per institution, as measured by either the number of sequence submissions to the database or the number of nucleotides sequenced (where the submitter’s institution information is known and recorded in the submitter fields in ENA). In the datasets we provide, these records are linked to specific submitting institutions per year.

The dataset of publications includes all data necessary to construct co-authorship networks of collaboration between individuals, institutions and countries that were involved in these sequencing efforts. Table 2 contains the figures for the total number of unique publications (PMIDs) we hold for each species (where the required information on author institutions could be automatically retrieved from SCOPUS) and the total number of institutions and countries involved in authoring these publications.

### Reflection on strengths and weaknesses

Our study reflects that the growing capacity in data infrastructure and the development of bioinformatics offers new opportunities not only for life scientists and molecular biology but also for social scientists and historians of science. The method outlined in this paper provides a novel source of evidence to evaluate the development and growth of collaboration in DNA sequencing and genomics research. It is also able to avoid placing a narrow focus on a number of key players based on previous studies or historical accounts. Our datasets show a diversity of countries and institutions involved in the sequencing of the human, yeast and pig genomes. Thus, they enable us to complement previous historical studies that have been focused on a limited number of large-scale sequencing centres (e.g. Hilgartner, 2017).

This analysis is, however, limited by the data infrastructure that we have used. Its organisation and, especially, its absences can indeed shape and affect how and what we can know about the past; how and what information is being recorded, what is missing, what can and cannot be automatically retrieved, what is considered important (or not), and for what questions the information was expected to provide answers to. These processes, including storage and curation, were built into the databases and can have significant impacts on what we know and what we can study about collaborations in genomic sequencing. For instance, in the ENA, a proportion of accession numbers did not have any further information about submitters. We need to consider these absences, along with their underlying meanings and power dynamics more carefully, especially when we use digital research methods and online data ( Leonelli, 2016; Lupton, 2015).

For this reason, we argue that qualitative work should accompany digital research methods. In our project, we are currently working on a mixed methods approach based on constant, bi-directional interactions between quantitative data and other qualitative historical evidence, such as documents stored in archives. This approach has been especially useful to highlight competing visions and different narratives of genomics, and how these might have changed over time.

## Data availability

### Underlying data

Edinburgh DataShare: Human, yeast and pig genomics: sequence submissions and first sequence descriptions in the literature (1980–2015). https://doi.org/10.7488/ds/2718 ( Wong *et al*., 2019).

This project contains the following underlying data:

- Human_publications.csv (Spreadsheet containing PMIDs and publication information for *Homo sapiens* sequences)
- human_submissions.csv (Spreadsheet containing institutional and submission information for *Homo sapiens* ENA submissions)
- Yeast_publications.csv (Spreadsheet containing PMIDs and publication information for *S. cerevisiae* sequences)
- yeast_submissions.csv (Spreadsheet containing institutional and submission information for *S. cerevisiae* ENA submissions)
- Pig_Publications.csv (Spreadsheet containing PMIDs and publication information for *Sus scrofa* sequences)
- pig_submissions.csv (Spreadsheet containing institutional and submission information for *Sus scrofa* ENA submissions)

Data are available under the terms of the Creative Commons Attribution 4.0 International license (CC-BY 4.0).

## Software availability

- Source code available from: https://github.com/UofGMarkWong/TRANSGENE
- Archived source code at time of publication: https://doi.org/10.5281/zenodo.3345686 ( UofGMarkWong, 2019)
- License: CC-BY 4.0
