# On the design of linked datasets mapping networks of collaboration in the genomic sequencing of *Saccharomyces cerevisiae*, *Homo sapiens*, and *Sus scrofa*

**DOI:** 10.12688/f1000research.18656.3

**Published:** 2023-02-28

**Authors:** Mark Wong, Rhodri Leng

**Affiliations:** 1 Urban Studies, School of Social and Political Sciences, University of Glasgow, Glasgow, G12 8QQ, UK; 2 Science, Technology and Innovation Studies, University of Edinburgh, Edinburgh, EH1 1LZ, UK

**Keywords:** Bibliometrics, Bibliographic Database, network analysis, genomics, S. cerevisiae, Homo sapiens, Sus scrofa, history of science

## Abstract

This data note describes a unique two-step methodology to construct six linked datasets covering the sequencing of *Saccharomyces cerevisiae*, *Homo sapiens*, and *Sus scrofa *genomes. The datasets were used as evidence in a project that investigated the history of genomic science. To design the datasets, we first retrieved all sequence submission data from the European Nucleotide Archive (ENA), including accession numbers associated with each of our three species. Second, we used these accession numbers to construct queries to retrieve peer-reviewed scientific publications that first described these sequence submissions in the scientific literature. For each species, this resulted in two associated datasets: 1) A .csv file documenting the PMID of each article describing new sequences, all paper authors, all institutional affiliations of each author, countries of institution, year of first submission to the ENA (when available), and the year of article publication, and 2) A .csv file documenting all institutions submitting to the ENA, number of nucleotides sequenced and years of submission to the database. We utilised these datasets to understand how institutional collaboration shaped sequencing efforts, and to systematically identify important institutions and changes in the structure of research communities throughout the history of genomics and across our three target species. This data note, therefore, should aid researchers who would like to use these data for future analyses by making the methodology that underpins it transparent. Further, by detailing our methodology, researchers may be able to utilise our approach to construct similar datasets in the future.

## Introduction

This data note describes the methodology used to construct six novel datasets for the European Research Council funded project, *Medical Translation in the History of Modern Genomics* (TRANSGENE); a project that explored the history of scientific collaboration around DNA sequencing. By investigating the interactions between different institutions in the determination and description of new DNA sequences, this project showed changing and varied configurations of genomic science between the 1980s and 2010s. These historical configurations and their dynamics were shaped by the objectives, organisation and development of research communities and their distinct target species ( García-Sancho & Lowe, 2023). To document this, the datasets captured sequencing practices specific to species that have been mobilised by a variety of scientists in different research contexts: a fungus with a strong tradition as a model organism and widespread use in the brewing and biotechnology industries (the baker’s yeast *Saccharomyces cerevisiae*), a farm animal that has been the object of agricultural genetics, immunogenetic research and commercial breeding programmes (the pig *Sus scrofa*), and *Homo sapiens* (subsequently referred to as ‘human’).

For each species, we systematically retrieved data related to sequence submissions to public databases and co-authorship relations underpinning the description of those sequences in the scientific literature. As part of the TRANSGENE project, we have stored all relevant datasets in the data repository at the University of Edinburgh (see *Data availability*; Wong *et al*., 2019).

In what follows, we first describe a unique two-step methodology that involved:

1. Extracting data on sequence submissions to the European Nucleotide Archive (ENA a public, open access database) via automated routines and Application Programme Interfaces (APIs).
2. Linking particular sequence submissions to peer-reviewed publications that first described these in the literature via API queries, which utilised sequence accession numbers to mine Europe PubMed Central and SCOPUS.

We then discuss our approach to re-structuring and cleaning these data and offer a description of the content of each dataset. Finally, we reflect on the strengths and weaknesses of these datasets and methods.

## Materials and methods

### Data collection

This project entailed a large and unique data collection exercise of over 13 million records, which were retrieved via 30 million API queries to three different databases. This involved a two-step process. First, we retrieved all sequence submission data from the ENA, including accession numbers associated with particular sequence lengths. Second, we used these accession numbers to construct API queries to retrieve peer-reviewed scientific publications that first described and linked to these sequence submissions in the scientific literature. 

### Extracting ENA submission data

We retrieved sequence submission data from the ENA for each of the three species over defined periods – *S. cerevisiae* (1980–2000), *H. sapiens* (1985–2005), and *S. scrofa* (1990–2015). The date ranges for each species were selected based on the history of science objectives underlying our project. The purpose was to capture submissions before, during, and after the completion of concerted efforts to comprehensively sequence the genome of each of the species. The search was conducted by making a series of calls to ENA’s API for each species and each year our project investigated. The query was constructed by specifying the taxon’s number (tax_eq) in the ENA index (i.e. 9606 for *H. sapiens*, 4932 for *S. cerevisiae* and 9823 for *S. scrofa*) and the sequence release date (first_public) to filter records that were released within a certain year. The search parameter of first_public was specified as “greater than or equal to” 1 ^st^ January and “less than or equal to” 31 ^st^ December of the year. Additional parameters were used to specify search for sequence release records ( result=sequence_release) and download the data in .XML format ( display=xml). In cases where records per year exceeded the ENA’s limit of 100,000 records per API call, the pagination function ( offset) was deployed.

This procedure allowed us to mine the ENA database based on the species and years relevant to our study and extract data on: 1) the number of nucleotides submitted for each of these species; 2) all accession numbers associated with these sequence lengths; 3) the date of submission; 4) the name of the submitting individual and/or their institutional affiliation (if available); and 5) papers in the scientific literature associated with each accession number (if specified by the submitter) ( Li *et al*., 2015). Further details about the API queries are contained in the R scripts made available together with the datasets (see *Software availability*; UofGMarkWong, 2019). As the ENA is part of The International Nucleotide Sequence Database Collaboration (INSDC), which facilitates the sharing of information of three main sequence databases, including the European Nucleotide Archive, based in the European Bioinformatics Institute, GenBank, provided by the US National Centre for Biotechnology Information (NCBI), and DNA Data Bank of Japan (DDBJ), we were able to retrieve all sequence submissions from institutions participating in these databases. Once collected, we utilised the R statistical environment ( R Development Core Team, 2016) to structure these data for further cleaning and analysis. In Table 1, we report the total records retrieved via this process.

**Table 1.** Total of ENA accession numbers and Europe PMC publication records retrieved for *S. cerevisiae*, *H. sapiens*, and *S. scrofa*.

*Species*	Total ENA submissions/ accession numbers (nucleotides)	Accession numbers that contain submitting individual records (nucleotides)	Accession numbers that contain publication records (nucleotides)	Accession numbers in which submitting individual or publication records were not found (nucleotides) *	PMIDs retrieved from Europe PMC
*S. cerevisiae* *(1980 – 2000)*	18,521 (32,726,254)	2,332 (14,420,456)	3,343 (11,517,249)	13,956 (14,413,985)	3,158
*H. sapiens* *(1985–2005)*	10,091,109 (21,034,707,659)	2,619,237 (16,942,665,389)	2,582,496 (10,466,788,214)	5,055,436 (3,996,992,385)	33,910
*S. scrofa* *(1990 – 2015)*	3,322,337 (18,890,916,045)	1,676,935 (10,275,568,002)	1,435,419 (2,969,582,959)	338,890 (8,174,825,230)	2,464

(*) *Despite not containing submitting individual records, some of these accession numbers were linked to a submitting institution. See Leng *et al.*, 2022: 288 (Table 1) for figures on accession numbers with institutional submitter information and how we used them to trace back sequencing practices in our investigation of the history of genomics.*

### Extracting Europe PMC and SCOPUS publication data

The submission data alone presented limitations to fulfill the objectives of the history of science project underlying our endeavor. Firstly, a substantial number of the accession numbers lacked submitter details and were thus not amenable to qualitative historical analysis: 75.35% of the yeast records, 50.1% of the human and 10.2% of the pig did not have a person’s name listed as the sequence submitter (see Table 1). Secondly, and especially in the pre-2000 accession numbers, the ENA often listed one submitter only, which was insufficient to investigate changing collaborative patterns. Due to this, we used publication and co-authorship data as a proxy to identify collaboration between different institutions involved in the description of the sequencing results.

We generated queries to the Europe PubMed Central’s (Europe PMC) API by using the ENA accession number as a parameter to search for associated publications ( EMBL_PUBS). This linkage allowed us to identify a list of PubMed IDs (PMIDs) of the publications linked to these accession numbers ( Lopez *et al*., 2014). In addition, other parameters were used including result_type=core to return full metadata available, format to download as .JSON files, cursorMark as a pagination option, and the default result limit of each API call ( pageSize) at 1000 publication records. We deployed a routine to automate the search for each accession number in our dataset. The routine’s procedure to compose and make an API call to Europe PMC using a list of accession numbers (pre-extracted using the ENA API call detailed) has been made available in an online repository (see *Software availability*; UofGMarkWong, 2019).

We then extracted fuller data on all authors, their institutional affiliations, the city and country of institution and the date of publication in SCOPUS using the PMIDs as a search parameter ( PMID) and utilising other default parameters such as apikey, apart from view=complete to specify returning of full meta-data. The routines and R scripts used are also available (see *Software availability*; UofGMarkWong, 2019). The use of two bibliometric databases was considered necessary, as while EuropePMC allowed searches for publications linked to and specifically describing an accession number, it only holds institutional information of the corresponding author for all records published before 2014 ( Europe PMC Consortium, 2015). SCOPUS, by contrast, holds fuller bibliometric details of all authors and their institutions, particularly for biomedical and natural science literature ( Rotolo & Leydesdorff, 2015). This was crucial for mapping inter-institutional collaboration. However, this database only allows searches based on its text-mining functions and returns publications that mention an accession number anywhere in the text – thus the necessity of inputting the PMIDs retrieved via EuropePMC that specifically describe the sequence submissions.

We selected only the first articles to be published associated with an accession number because first publications are more likely to be written by the groups responsible for the submission of the original version of the sequence (either to describe their contribution or to use it in agricultural or biomedical research). Although this correspondence between submission and first publication was not universal, ^1^ our search strategy excluded papers that utilised particular sequence lengths that had already been described in the literature and consequently refined our corpus of PMIDs (see differences between Table 1 and Table 2).

**Table 2.** Total number of PMIDs, institutions, and countries extracted from SCOPUS for *S. cerevisiae* (1980–2000), *H. sapiens* (1985–2005) and *S. scrofa* (1990–2015).

Species	PMIDs	Institutions	Countries
*S. cerevisiae* (1980–2000)	2,887	685	39
*H. sapiens* (1985–2005)	24,726	6,014	101
*S. scrofa* (1990–2015)	1,947	1,272	64

### Data cleaning and description

Once collected, researchers in our team cleaned these datasets via VantagePoint (2017) v.10 by using a combination of fuzzy logic algorithms available in the software (i.e. “Fuzzy word matching” to make word comparisons at 95% or lower) and manual cleaning to standardise institution, author and country names according to a pre-specified protocol. The protocol specified ensured consistency in name conventions, fully spelling out acronyms and abbreviations, removing articles and legal entities, using proper case conventions, removing white spaces and ineligible characters, removing duplicates, and keeping school and department data if it appeared more than 50 times in the dataset. All our publication dataset and the main submitting institutions, as documented by the volume of DNA nucleotides registered in the ENA, were cleaned according to this protocol. Missing data, particularly regarding institutional affiliation, was filled manually by scrutinising the record on SCOPUS’ web front-end. To replicate this cleaning process, other open source software, such as OpenRefine, may also be used as an alternative.

In total, each species has two associated datasets: 1) A .csv file documenting the PMID of each article describing new sequences, all paper authors, all institutional affiliations of each author, countries of institution, year of first submission to the ENA (when available), and the year of article publication, and 2) A .csv file documenting all institutions submitting to the ENA, number of nucleotides sequenced and years of submission to the database. While the data about yeast submissions is provided sequence per sequence with full dates and information about both submitting individuals and institutions, the pig and human submission datasets offer aggregate figures of both number of sequence submissions to the database and number of nucleotides sequenced per institution per year.

Our corpus of publications includes all data necessary to construct co-authorship networks of collaboration between individuals, institutions and countries that were involved in the sequencing efforts. Table 2 contains the figures for the total number of publications (PMIDs) that each dataset holds for our target species and the total number of institutions and countries involved in authoring these publications. Within our project, we built three networks visualising co-authorship relations between institutions describing new human, yeast and pig DNA sequences in the scientific literature and used them as evidence to investigate the history of genomics ( Leng *et al.,* 2022: 301ff).

### Reflection on strengths and weaknesses

Our study reflects that the growing capacity in data infrastructure and the development of bioinformatics offers new opportunities not only for life scientists and molecular biology but also for social scientists and historians of science. The method outlined in this paper provides a novel source of evidence to evaluate the development and growth of collaboration in DNA sequencing and genomics research. It is also able to avoid placing a narrow focus on a number of key players based on previous studies or historical accounts. Our datasets show a diversity of countries and institutions involved in the sequencing of the human, yeast and pig genomes. Thus, they enable us to complement previous historical studies that have been focused on a limited number of large-scale sequencing centres (e.g. Hilgartner, 2017; Stevens, 2013).

This analysis is, however, limited by the data infrastructure that we have used. Its organisation and, especially, its absences can indeed shape and affect how and what we can know about the past; how and what information is being recorded, what is missing, what can and cannot be automatically retrieved, what is considered important (or not), and for what questions the information was expected to provide answers to. These processes, including storage and curation, were built into the databases and can have significant impacts on what we know and what we can study about collaboration in genomic sequencing. For instance, as noted above, a substantial proportion of accession numbers in the ENA did not have any further information about submitters. We need to consider these absences, along with their underlying meanings and power dynamics more carefully, especially when we use digital research methods and online data ( Leonelli, 2016; Lupton, 2015).

For this reason, we argue that qualitative work should accompany digital research methods. In our project, we developed a mixed methods approach based on constant, bi-directional interactions between quantitative data and other qualitative evidence, such as documents stored in archives ( García-Sancho & Lowe, eds., 2022). This approach has been especially useful to highlight competing visions and different historical configurations of genomics that have changed over time and across species and research communities ( García-Sancho & Lowe, 2023).

## Data Availability

Edinburgh DataShare: Human, yeast and pig genomics: sequence submissions and first sequence descriptions in the literature (1980–2015). https://doi.org/10.7488/ds/2718 ( Wong *et al*., 2019). This project contains the following underlying data: Human_publications.csv (Spreadsheet containing PMIDs and publication information for *H. sapiens* sequences) human_submissions.csv (Spreadsheet containing information for *H. sapiens* ENA sequence submissions) Yeast_publications.csv (Spreadsheet containing PMIDs and publication information for *S. cerevisiae* sequences) yeast_submissions.csv (Spreadsheet containing information for *S. cerevisiae* ENA sequence submissions) Pig_Publications.csv (Spreadsheet containing PMIDs and publication information for *S. scrofa* sequences) pig_submissions.csv (Spreadsheet containing information for *S. scrofa* ENA sequence submissions) Data are available under the terms of the Creative Commons Attribution 4.0 International license (CC-BY 4.0).

## Footnotes

^1^ It was beyond our means to verify the correspondence of authors in the over 4 million accession numbers associated to a scientific publication. However, in a sample that represented each of the 21 years comprising the yeast and human datasets (1980 to 2000 and 1985 to 2005) and each of the 26 years comprising the pig dataset (1990 to 2015) we found that in 62 of the 68 accession numbers there was a match between at least one of the authors listed in the first publication describing the sequence and the submitter details. This match occurred in all the 21 human records, 25 of the 26 pig records and 16 of the 21 yeast records of our sample. We also found that publications describing one single accession number dramatically decrease after 1992 across the three species and that, from the mid-1990s onwards, a growing proportion of accession numbers list more than one submitter. Records close to the years in which whole-genome sequencing projects are completed for each species tend not to have associated submitter or publication data. For full details of our sample analysis, see https://www.pure.ed.ac.uk/ws/portalfiles/portal/321338087/dna_submission_analysis.pdf

## Software availability

- Source code available from: https://github.com/UofGMarkWong/TRANSGENE
- Archived source code at time of publication: https://doi.org/10.5281/zenodo.3345686 ( UofGMarkWong, 2019)
- License: CC-BY 4.0
