# Supplementary material for: On the design of linked datasets mapping networks of collaboration in the genomic sequencing of Saccharomyces cerevisiae, Homo sapiens, and Sus scrofa
Source: F1000Res. 2023 Feb 28;8:1200. Originally published 2019 Jul 26. [Version 3] doi: 10.12688/f1000research.18656.3 (PMC7871417; doi:10.12688/f1000research.18656.3)
Supplement: Peer review history

# Peer review: Reviewer response for version 2

**DOI:** 10.5256/f1000research.30314.r77959

**Published:** 2021-02-08

**Authors:** Jake Lever

**Affiliations:** 1 Department of Bioengineering, Stanford University, Stanford, CA, 94305, USA

I would like to thank the authors for this read. It was a thorough and clear explanation of valuable methods for linking institutional and authorship metadata with submitted sequence data to the European Nucleotide Archive. Good clean metadata is the dream of all bioinformaticians, and public archives often fall short. This work is a nice illustration of linking together multiple public archives with a lot of manual effort to build a valuable metadata dataset for an interesting historical project.

 I have some minor comments and one request that I hope will not be too laborious.

1. This is valuable work and I feel the abstract sells it short. Firstly, I would recommend a clearer problem statement or sentence about the larger project before diving into the methods. Also, you don't make enough of why you need to link in EuropePMC and SCOPUS. Some quick statistics on the number of ENA accessions missing the needed metadata would really illustrate why the link to the literature is such a good idea. i.e. 50% of Homo sapiens accessions are missing both submitted and publication metadata
2. I assume it's due to the scope of the larger project, but a sentence mentioning why the choice of the three species would be nice.
3. I'd just like to applaud the sharing of the code. Yay for open science.
4. My one request that might take a few hours of work but would be a valuable addition: Your method uses the first publication that is linked to an accession as likely being the original authors. That is probably right. But a small validation would be really useful here. You could pick 10 (or more) random accession and see if that strategy worked. It doesn't have to be perfect, but good to see if it works okay.

Are sufficient details of methods and materials provided to allow replication by others?

Yes

Is the rationale for creating the dataset(s) clearly described?

Yes

Are the datasets clearly presented in a useable and accessible format?

Yes

Are the protocols appropriate and is the work technically sound?

Yes

Reviewer Expertise:

Bioinformatics & biomedical machine learning

I confirm that I have read this submission and believe that I have an appropriate level of expertise to confirm that it is of an acceptable scientific standard.

# Peer review: Reviewer response for version 1

**DOI:** 10.5256/f1000research.20425.r61097

**Published:** 2020-06-03

**Authors:** Rachel Ankeny

**Affiliations:** 1 Departments of History and Philosophy, University of Adelaide, Adelaide, SA, Australia

This article describes methodologies used to construct datasets on sequence submissions and co-authorship relationships relating to genomic sequencing of three major organisms. The methodology is clearly described, as are its limitations and prospects for use by other scholars. I particularly appreciated the careful reflections on strengths and weaknesses of the approaches taken, and agree that these approaches have clear prospects for enriching our historical/sociological accounts given tendencies to focus on the strongest (or loudest!) research centres to the neglect of other participants particularly in genomic sequencing efforts. Would strongly suggest citing Leonelli's book ^1^ on data curation (in addition to Lupton's more general work on this topic) as it addresses many of the relevant issues raised in the limitations relating to curation and completeness of data specifically in the context of modern biology.

Are sufficient details of methods and materials provided to allow replication by others?

Yes

Is the rationale for creating the dataset(s) clearly described?

Yes

Are the datasets clearly presented in a useable and accessible format?

Yes

Are the protocols appropriate and is the work technically sound?

Yes

Reviewer Expertise:

History/philosophy of contemporary biological sciences (hence I am not qualified to assess the details of the methodologies utilised in terms of data science but have expertise in use of larger datasets for historical explorations)

I confirm that I have read this submission and believe that I have an appropriate level of expertise to confirm that it is of an acceptable scientific standard.

## References

[1] : Data-Centric Biology. *University of Chicago Press* .2016;

# Response

**Published:** 2020-10-18

**Authors:** Mark Wong

**Affiliations:** University of Glasgow, UK

Thank you for your positive comments and excellent feedback. We are grateful for your time and appreciate your comments on the originality, significance, and timeliness of this paper. We have adopted your suggestion and cited Leonelli's (2016) seminal work in this area too.
